# Mind the gap: a nationwide survey study of undergraduate medical communication curricula in Italy

**DOI:** 10.1186/s12909-025-08522-8

**Published:** 2026-03-09

**Authors:** Roberta Martina Zagarella, Sarah Bigi, Melissa Nava, Chiara Midea, Sibilla Parlato, Serena Barello

**Affiliations:** 1 Interdepartmental Center for Research Ethics and Integrity, National Research Council, Via dei Taurini 19, Rome, 00185 Italy; 2 Department of Foreign Languages and Literatures, Catholic University of the Sacred Heart, Milan, Italy; 3 WHYpsy Lab, Department of Brain and Behavioural Sciences, University of Pavia, Pavia, Italy

**Keywords:** Healthcare communication, Clinical communication education, Curriculum mapping, Medical curricula, Italy, Culturally adaptive training, Patient-centered care

## Abstract

**Background:**

Effective clinical communication is essential to safe, patient-centered care, yet its integration into undergraduate medical curricula remains inconsistent worldwide. In Italy, no national-level evidence has been available to guide reform in line with local specificities. This study aimed to systematically map and analyze healthcare communication education in Italian medical and health professions programs, identifying strengths, gaps, and opportunities for innovation.

**Methods:**

The ComMedInItaly study adopted a two-phase design: (1) systematic mapping of undergraduate healthcare communication courses across accredited Italian universities; and (2) a national survey of course instructors exploring content, pedagogical approaches, assessment methods, and instructor expertise. Findings from both phases were descriptively analyzed and integrated.

**Results:**

Twenty-four courses were identified, most positioned in the early years of study, assigned limited credits, and often embedded in psychology modules. Teaching was predominantly lecture-based, with limited use of experiential learning or performance-based assessment. Course content emphasized relational and psychological aspects, while interactional and linguistic dimensions were underrepresented. Instructor profiles were heterogeneous, with few affiliations to professional societies in healthcare communication.

**Conclusions:**

Healthcare communication training in Italy is fragmented, under-resourced, and weakly connected to clinical practice.

This is the first national-level analysis of communication education in Italy, establishing an empirical baseline for reform. The study introduces a replicable two-phase model, demonstrates the need for locally generated and culturally responsive evidence, and offers a pathway for aligning international frameworks with national realities. Together, these innovations position communication as a core, measurable competency in medical education.

**Supplementary Information:**

The online version contains supplementary material available at 10.1186/s12909-025-08522-8.

## Background

The importance of teaching communication skills to future physicians has long been recognized in medical education. Effective communication is essential for accurate diagnosis, therapeutic adherence, patient satisfaction, and clinical safety. Yet, despite this consensus, studies consistently highlight the need to strengthen communication training in undergraduate curricula [1–6].

International initiatives have sought to formalize and promote communication education. The Kalamazoo Consensus Statement [7] provided a structured framework of core communication tasks, later expanded by the Glasgow Consensus Statement [8], which emphasized the role of communication in reinforcing the humanity of healthcare and called for integration, interdisciplinarity, and accountability at the system level. While many countries have advanced in embedding communication into curricula, reforms remain uneven, shaped by accreditation standards, cultural expectations of the doctor–patient relationship, and institutional recognition of communication as a professional competency.

Italy offers a particularly compelling case. The country combines a strong biomedical tradition with growing policy attention to patient-centered care, yet structural barriers persist in embedding communication into medical education. This paradox not only illuminates challenges faced by systems with similar traditions but also offers transferable lessons for strengthening curricula internationally.

Translating international recommendations into practice requires attention to local sociocultural and institutional contexts. Communication training is influenced by national education systems, healthcare delivery models, and cultural norms surrounding the doctor–patient relationship. Silverman and colleagues [9] argue that developing communication competence requires longitudinal, clinically integrated, and experiential approaches. However, such models often encounter resistance in contexts where biomedical knowledge is prioritized over interpersonal competence. In Italy, as in other countries, communication is still frequently perceived as a “soft skill” rather than a core component of clinical identity and effectiveness [4, 5].

Scholars further caution against reducing communication to standardized techniques. Rossi and Sarangi [10] stress that this risks neglecting the interpretive, relational, and ethical dimensions of clinical meaning-making. Echoing critiques from the medical humanities, communication should be understood as a co-constructed process embedded in empathy, trust, and power dynamics [3, 6]. Without this broader framing, communication curricula risk failing to prepare students for the complex realities of clinical encounters.

Although interest in healthcare communication has grown in Italy over the past decade, available studies focus largely on isolated initiatives or postgraduate settings. No national survey has systematically mapped the undergraduate training landscape. This gap limits the ability to identify inequities, share best practices, and design reforms that are evidence-informed and context-sensitive.

To address this gap, the “Communication in Medical Curricula in Italy” (ComMedInItaly) study was developed to provide the first comprehensive overview of healthcare communication education in Italian Schools of Medicine. Using a combination of curriculum mapping and a national survey of instructors, the study explores the structure, content, and pedagogical approaches of existing courses, with particular attention to how teaching aligns with international models and national needs. In doing so, it generates the first empirical baseline for reform and introduces an innovative, replicable model for designing context-sensitive and culturally responsive communication curricula that can inform both national and international reforms.

## Materials and methods

This study employed a two-phase mixed-methods design to examine healthcare communication education in Italian undergraduate medicine and health profession programs.

Phase 1 consisted of a systematic mapping of accredited university websites to identify communication-related courses. Data extracted from official curricula and syllabi included course title, year of delivery, credit allocation, disciplinary classification, instructor identity, pedagogical approaches, and assessment methods, providing a national baseline of course availability and structural characteristics.

Phase 2 involved a structured online survey administered to instructors identified in Phase 1. The survey explored course objectives, content domains, teaching strategies, assessment techniques, and instructor profiles, including expertise in healthcare communication.

Integrating curricular mapping with instructor-reported data enabled triangulation and yielded a comprehensive picture of strengths and gaps in current provision. The study was conducted by an interdisciplinary team with expertise spanning medical education, clinical communication, linguistics, psychology, philosophy of language, and ethics, ensuring a multi-perspective analysis grounded in both pedagogical and practice-oriented considerations.

### Course mapping

We systematically mapped healthcare communication courses offered across Italian universities, encompassing both Schools of Medicine and programs in the health professions, to provide a comprehensive overview of communication education across disciplines involved in patient care. The initial sampling frame comprised Schools of Medicine in major Italian cities, later expanded to include diverse faculties and degree programs.

Curricula were reviewed for programs in Medicine and Surgery, Dentistry, Nursing, Obstetrics/Midwifery, Orthopedics, Ophthalmology, Physiotherapy, Dental Hygiene, Rehabilitation Sciences, Dietetics, Healthcare Assistance, Neurophysiopathology, Cardiophysiopathology, Technical and Diagnostic Healthcare Sciences, and Podology.

Data collection was conducted manually by consulting the official websites of each academic program. No software tools were used. The criteria for collection and assembly of data were determined by the research group before the beginning of data collection and then rediscussed when doubts arose regarding the inclusion or exclusion of certain information. Study plans and, where available, course syllabi were examined to identify educational offerings related to healthcare communication. Relevant courses were detected using targeted keyword searches (e.g., “communication,” “communicative,” “interaction,” “doctor-patient relationship”).

For each identified course, we extracted data on: academic credits, year of delivery within the curriculum, status as a stand-alone course or module, disciplinary classification within the Italian Scientific Disciplinary Sectors/Groups^1^, instructor names and affiliations, and –when available – syllabus details on learning objectives, teaching methods, and assessment strategies.

### Instructor survey

Based on the initial mapping results, the second study phase comprised the design and administration of a structured online survey targeting instructors responsible for healthcare communication courses in Italian undergraduate medicine and health profession programs. The survey aimed to characterize current teaching practices, examining course content, pedagogical approaches, assessment strategies, and the alignment between instructors’ disciplinary expertise and course material. The survey was not piloted and we did not assess psychometric properties, as the instrument was not designed to function as a psychometric scale. The questionnaire was adapted from the survey developed by Ury et al. (2003) [11] to assess communication training in medical education, and in this study it was used exclusively for descriptive, curriculum-mapping purposes. Items captured factual information (e.g., course structure, teaching methods, assessment approaches) rather than underlying latent constructs, making reliability or construct validity testing conceptually not aligned with the survey purposes. For these reasons, formal psychometric evaluation was not undertaken.

The target population included all instructors identified in the mapping phase (≈ 60), each invited via institutional email (publicly available through university websites). Instructors responsible for multiple courses were asked to complete one entry per course. The 28-item questionnaire, administered in Italian, required approximately 15 min to complete and included multiple-choice and open-ended questions, with “Other” options for alternative responses.

The questionnaire was organized into five thematic sections: (1) instructor and course profile (e.g., socio-demographic characteristics, academic rank, disciplinary background, teaching experience, course features, language of instruction, and scientific society memberships); (2) course objectives and targeted communication skills; (3) teaching methodologies; (4) assessment practices and perceived effectiveness; and (5) perspectives on improvement and future development needs (see Appendix A).

### Survey platform

The questionnaire was administered via EUSurvey^2^, the European Commission’s open-source platform, which ensures robust data security and GDPR compliance^3^.

The survey was conducted using EUSurvey’s “Advanced Privacy” mode, ensuring full anonymity by preventing access to connection data (e.g., IP addresses, metadata). Only essential operational cookies were enabled for secure survey completion, with no profiling or tracking cookies. Participants were informed of the cookie policy and could decline all non-essential cookies. Operational cookies were used solely for security purposes and retained only for the minimum time required to maintain system integrity and comply with data protection regulations.

### Ethical issues

The study was conducted in accordance with established ethical standards and in compliance with national and European data protection regulations. The research protocol received formal approval from the Research Ethics and Integrity Committee of the National Research Council^4^ (CNR) (Ref. 0171973/2024).

No personally identifiable information was collected, and all survey items were designed to minimize the risk of direct identification. Although the possibility of indirect identification could not be fully excluded due to the limited target population, participants were informed of this minimal residual risk. They were advised that any inadvertently disclosed personal details in open-ended responses would be removed during data processing to preserve anonymity. Re-identification was explicitly excluded from the study’s scope, and the research team committed not to attempt it. Before starting, participants received an information sheet detailing the study’s objectives, voluntary nature of participation, data types collected, and withdrawal procedures. Informed consent was obtained electronically via a “YES” confirmation; given the anonymous design, submitted responses could not be withdrawn or linked to individual identities.

## Results

The findings of the ComMedInItaly study are based on two complementary data sources: the systematic mapping of Italian university websites and the targeted survey addressed to instructors of healthcare communication courses. This combined approach allowed us to capture both the structural organization of communication training and the perspectives of those directly involved in teaching it. The mapping revealed widespread trends, which were corroborated by the survey responses, offering a picture of the current state of communication education in Italian medical schools.

The mapping phase revealed 24 courses across Italian universities that met the study’s inclusion criteria. The online survey was distributed to 53 instructors identified in the mapping; some courses were held by more than one instructor. A total of 18 survey responses were received, of which 15 were confirmed to be from instructors teaching a healthcare communication course. Three responses were excluded in accordance with the inclusion criterion requiring participants to be actively responsible for a course at the time of the survey completion. The 15 valid responses – representing approximately 28% of the invited sample – were retained for analysis.

While the limited sample size precludes statistical generalizability, the consistency between the respondents’ profiles and the findings from the national course mapping lends credibility to the descriptive results. These findings offer valuable insights for informing future research and guiding the development of targeted curricular improvements in healthcare communication education.

### Instructors’ profile

As shown in Table 1, of the 15 instructors who completed the survey, 60% identified as female and 40% as male. The majority of respondents (73%) were between 51 and 66 years of age, while an additional 20% were aged 67 years or older. Regarding academic position, 40% held the role of adjunct professor, with the remaining respondents evenly distributed between associate professors (20%) and full professors (20%).

The respondents represented a range of disciplinary backgrounds, most commonly general psychology, followed by clinical psychology, pedagogy, history of medicine, language and communication sciences, and clinical disciplines. Notably, 67% of instructors reported more than 10 years of experience teaching healthcare communication. However, only a minority (20%) indicated formal affiliation with scientific societies specifically dedicated to healthcare communication.

**Table 1:** Instructors’ profile

Gender	60% female; 40% male
Age distribution	73% aged 51–66; 20% ≥67
Academic position	40% adjunct; 20% associate; 20% full professor
Disciplinary backgrounds	General psychology; clinical psychology; pedagogy; history of medicine; language and communication sciences; clinical disciplines
> 10 years teaching experience	67%
Affiliation with communication societies	20%

### Courses’ structure

The structural characteristics of healthcare communication courses identified through the national curriculum mapping were consistent with the patterns reported by survey respondents (see Table 2). These courses are predominantly offered in central and northern regions of Italy, with a notable concentration in the Lombardy region. The vast majority (80%) are delivered in Italian, reflecting the linguistic context of medical training in the country.

Communication training is typically introduced during the early stages of healthcare education, most often in the first year of the curriculum. This positioning suggests a foundational role but may also limit the integration of communication skills with clinical experience. Across programs, healthcare communication is delivered either as short, integrated modules within broader courses or as stand-alone courses. Regardless of format, these offerings tend to be under-resourced in terms of academic credit allocation, with most courses receiving between 1 and 3 European Credit Transfer and Accumulation System (ECTS) credits.

Notably, the highest number of academic credits allocated to communication-related coursework was observed in health professions programs – particularly in the degree program in Obstetrics at the Università Vita-Salute San Raffaele (Lombardy region), where a course entirely dedicated to communication (“La Comunicazione”) awards 8 ECTS credits and spans multiple disciplinary sectors (M-PSI/08 Clinical Psychology, M-PSI/01 General Psychology, M-PED/01 Pedagogy, Theories of Education and Social Education, MED/47 Midwifery). This represents a rare example of substantial curricular investment in communication training within the Italian academic context.

In contrast, most programs address communication within the framework of broader modules – typically in courses such as Clinical and General Psychology. While these courses provide valuable psychological foundations and may introduce basic interpersonal strategies for clinician-patient interactions, they often lack a dedicated focus on the interactional and linguistic dimensions of communication.

Interestingly, the course mapping also revealed that healthcare communication is occasionally addressed within English-language modules, particularly in courses labeled “Clinical English.” In these courses, students are introduced to models of doctor-patient interaction in English, often with the dual aim of developing both linguistic and communicative competencies for clinical practice in international or multilingual contexts. Among the reference frameworks adopted, the Calgary-Cambridge Model [9, 12] appears to be the most frequently cited.

Communication is also partially incorporated into courses titled “Scientific English,” which primarily aim to equip students with the lexical and grammatical knowledge required for professional communication in a foreign language. However, these courses occasionally include components that address communicative strategies for interacting with non-native speakers or international patients. A commonly cited model in this context is the SPIKES protocol [13], which provides a structured approach for delivering bad news in clinical settings.

The inclusion of communication content in other areas – such as “Scientific English” or “Clinical English” – further reflects a fragmented approach, where communicative competence is not always framed as a core clinical skill but rather as a peripheral or instrumental component. This variability underscores the need for greater clarity and standardization in how communication is conceptualized and integrated across healthcare curricula.

**Table 2:** Courses’ structure

Geographical distribution	Mostly central and northern Italy; concentration in Lombardy
Language	80% Italian
Year of delivery	Typically year 1
Course format	Short modules within broader courses or stand‑alone
Credit allocation	Most courses 1–3 ECTS; rare high‑credit examples (e.g., 8 ECTS in Obstetrics at Università Vita‑Salute San Raffaele)
Common host courses	Clinical Psychology; General Psychology; Clinical English; Scientific English
Reference frameworks	Calgary‑Cambridge Model; SPIKES protocol

### Course objectives and methods

The primary objectives most frequently reported by instructors were to promote knowledge and understanding of core communication competencies in clinical practice – particularly patient-centered communication –, to foster positive attitudes toward effective communication in medicine, and to encourage the deliberate use of communication as a therapeutic tool.

In terms of course content, the most commonly addressed topics included communication during diagnostic and treatment processes and emotional management in patient interactions, each cited by 87% of respondents in a multiple-response item. Special emphasis was placed on the delivery of bad news, with many courses incorporating specific protocols (e.g., SPIKES) or exercises aimed at preparing students to interact with vulnerable or complex patient populations.

When asked to identify the most critical aspects to highlight in communication training, respondents prioritized the emotional dimension of clinical encounters and the role of nonverbal behaviors – such as gestures, posture, and eye contact – followed by contextual factors like timing and setting.

The analysis of teaching materials, based on university website mapping, revealed considerable heterogeneity. There appears to be no standardized set of reference materials (e.g., handbooks or peer-reviewed articles), and the majority of recommended readings are in English. A broad range of disciplinary perspectives is represented, with most course materials authored by psychologists or clinicians. In many cases, instructors rely on slide presentations or assign readings on the doctor-patient relationship and the general psychology of communication.

Regarding instructional methods, traditional lectures remain the most widely used format, reported by 80% of survey respondents, followed by group work (73%) and video analysis of patient-provider interactions (67%). A variety of other pedagogical approaches were also reported – albeit less frequently – including role-playing and simulations, discussion of real clinical cases, seminar-style debates, multimedia content (e.g., video clips, films), and storytelling by practicing physicians.

Assessment practices are equally diverse. Common methods include oral and written examinations, often comprising open-ended or multiple-choice questions. In some instances, mere attendance suffices for course completion. More interactive or competency-based evaluation techniques – such as simulated clinical scenarios, peer-to-peer assessments, and direct observation of patient interactions – were reported in only a minority of cases. The perceived effectiveness of communication training was most frequently assessed via student feedback, followed by instructor observation and specific skill testing.

Finally, respondents identified several key areas for future improvement: the integration of new technologies into teaching (47%), regular updating of course content to reflect evolving best practices (40%), and reconsideration of the course’s timing and placement within the broader curriculum (33%). See Table 3 for an overview of these findings.

**Table 3:** Course objectives, content, and methods

Primary objectives	Promote knowledge and understanding of core communication competencies in clinical practice; foster positive attitudes towards effective communication in medicine; encourage the deliberate use of communication as therapeutic tool
Primary Topics Covered	Communication during diagnostic and treatment processes; emotional management in patient interactions; bad‑news delivery
Critical aspects emphasized	Emotional dimension of clinical encounters; nonverbal communication; contextual factors (e.g. timing and setting)
Teaching materials	No standard references; mostly English readings; psychological/clinical sources
Teaching methods	Mostly lectures, group work and video analysis of patient-provider interactions. Limited use of role-playing, simulation, discussion of real clinical cases, storytelling, etc.
Assessment methods	Oral/written exams; attendance. Limited use of simulations, peer-to-peer assessments, direct observation of interactions
Areas for improvement	Integration of new technologies into teaching; regular content updating; course timing/placement

## Discussion

The ComMedInItaly study highlights substantial gaps in the current provision of healthcare communication education in Italian undergraduate programs. Despite international consensus on its centrality to safe, effective, and patient-centered care [7, 8], communication training in Italy remains fragmented, under-resourced, and weakly integrated into curricula.

A first concern is the marginal positioning of communication courses. Typically offered in the earliest years of study and allocated only one to three ECTS credits, such provision risks divorcing communication learning from clinical experience. Students often lack the practical grounding to engage with the complexity of real encounters, a structural misalignment also reported in other European and Asian contexts.

Instructor profiles further reflect this lack of institutional coherence. Courses are frequently taught by internal resources drawn from psychology, humanities, or clinical backgrounds, but without clear standards for recruitment or affiliations with professional societies. In some cases, instructors from business or marketing disciplines teach communication, framing patients as “customers” – a perspective at odds with patient-centered care.

Course content tends to emphasize relational and psychological skills, such as empathy and active listening, while underrepresenting interactional and linguistic competencies that are essential for managing dialogue, negotiating meaning, and supporting shared decision-making. This imbalance is not unique to Italy: internationally, curricula risk reducing communication to “soft skills” rather than recognizing it as a structured and professional domain.

Pedagogically, Italian programs remain dominated by traditional lectures. Experiential and interactive approaches – role-play, simulation, standardized patient encounters, reflective practice – are supplementary rather than central. Yet international evidence consistently demonstrates that such methods are most effective for developing communication competence [9, 12, 14, 15].

Assessment practices show a similar misalignment: reliance on written or multiple-choice exams contrasts with more authentic, performance-based evaluations, such as Objective Structured Clinical Examinations (OSCEs), or simulated encounters, used in countries with more mature curricula [16, 17].

Finally, the absence of a formal disciplinary designation for healthcare communication within the Italian Scientific Disciplinary Sectors mirrors challenges faced in other contexts. Without institutional recognition, it is difficult to establish curricular standards, define faculty qualifications, or secure sustainable resources, leaving communication education fragmented and undervalued.

Taken together, these findings reflect systemic barriers that prevent communication from being embedded as a core clinical competency. At the same time, they illustrate broader international challenges in professionalizing communication education. Addressing them requires not only incremental improvements but also systemic reform guided by robust evidence. By providing the first comprehensive map of Italian curricula, the ComMedInItaly study contributes precisely this kind of evidence, creating the conditions for reform that is both context-sensitive and aligned with international best practice.

## Conclusions

The Italian case provides a valuable lens for understanding the challenges of embedding communication as a core competency in medical education. The findings of this study reflect broader international trends in countries with strong biomedical traditions but limited emphasis on interpersonal competencies. Overall, healthcare communication education in Italy appears fragmented, inconsistently integrated, and undervalued, highlighting the need for systemic reform rather than isolated initiatives.

Beyond identifying national gaps, the study introduces a scalable and replicable model for systematically mapping communication curricula. This approach is innovative because, without robust data, meaningful reflection and evidence-based reform remain impossible. Replicating the model in other countries would allow for the development of a comparative evidence base, strengthening international collaboration and curricular alignment.

The results also underline the importance of culturally sensitive approaches. Communication practices are not universal: in contexts where patient autonomy is prioritized, communication supports informed decision-making, whereas in more hierarchical or collectivist settings, patients often defer to professional authority. Training programs must therefore adapt to cultural and linguistic specificities while fostering empathy, trust, and equity in line with universal ethical standards [18].

From an implementation science perspective, the ComMedInItaly model demonstrates how empirical data can be translated into practical interventions to guide curricular reform. In the Italian case, this means moving toward nationally coordinated guidelines, longitudinal integration of communication training across the medical pathway, investment in faculty development, and formal recognition of healthcare communication as an academic discipline. Ultimately, the innovation of this study lies in showing how locally generated evidence can transform international frameworks into context-sensitive reforms, offering a model for advancing healthcare communication as a globally relevant but culturally adaptable core of medical education [3, 19, 20].

Improving communication competencies at the workforce level is not merely an educational priority but a public health imperative. As underscored in the pEACH position paper on healthcare communication during COVID-19 [21], deficits in communication capacity can exacerbate misinformation, erode trust, and undermine adherence to preventive and therapeutic measures, particularly in times of crisis. Conversely, well-trained professionals can play a pivotal role in delivering clear, consistent, and culturally sensitive messages, supporting citizen engagement, and reducing inequities in access to information and care.

By aligning reforms in healthcare communication education with internationally endorsed frameworks and public health goals, Italy can enhance both individual patient outcomes and population-level health indicators, positioning communication as a strategic lever for equity and system sustainability. Our findings point to several priority actions for Italy and comparable contexts: strengthening national coordination to ensure curricular equity, professionalizing faculty training, expanding experiential and reflective pedagogy, introducing authentic assessment methods, and embedding communication longitudinally within clinical learning.

Taken together, these directions provide not just a roadmap for reform but also evidence of how locally generated data can drive globally relevant innovation. The ComMedInItaly study demonstrates that advancing healthcare communication requires moving beyond generic recommendations to context-sensitive, evidence-based strategies – offering a model for integrating communication as a visible, measurable, and culturally adaptable dimension of medical education and public health resilience.

## Supplementary Information

Supplementary Material 1. (Appendix A).

## Data Availability

Aggregated and anonymized data supporting the findings of this study are available from the corresponding author on reasonable request.

## Footnotes

1 Scientific Disciplinary Group (Gruppi Scientifico‑Disciplinari - GSD) are official categories defined by the Italian Ministry of University and Research to classify and organize academic disciplines for teaching, research, and recruitment purposes in universities. At the time of our study, Italian universities still operated under the traditional Scientific Disciplinary Sectors (Settori Scientifico‑Disciplinari - SSD) system. However, with the implementation of Ministerial Decree No. 639 of 2 May 2024, those SSDs were reorganized into the new Gruppi Scientifico‑Disciplinari (GSD). Cf. https://www.mur.gov.it/it/aree-tematiche/universita/docenti-universitari-e-carriera-accademica/settori-concorsuali-e-settori.

2
https://ec.europa.eu/eusurvey/home/welcome.

3 For more information on data processing by the EUSurvey platform: https://ec.europa.eu/eusurvey/home/privacystatement?language=it; https://ec.europa.eu/info/cookies_it.

4
https://www.cnr.it/en/ethics.

## References

[1] Hargie O, Dickson D, Boohan M, Hughes K. A survey of communication skills training in UK schools of medicine: present practices and prospective proposals. Med Educ. 1998;32:25–34. 10.1046/j.1365-2923.1998.00154.x. 9624396 10.1046/j.1365-2923.1998.00154.x

[2] Al Odhayani A, Ratnapalan S. Teaching communication skills. Can Fam Physician. 2011;57:1216–8. 21998240 PMC3192093

[3] Bachmann C, Abramovitch H, Barbu CG, Cavaco AM, Elorza RD, Haak R, et al. A European consensus on learning objectives for a core communication curriculum in health care professions. Patient Educ Couns. 2013;93:18–26. 10.1016/j.pec.2012.10.016. 23199592 10.1016/j.pec.2012.10.016

[4] Wouda JC, van de Wiel HBM. Education in patient-physician communication: how to improve effectiveness? Patient Educ Couns. 2013;90:46–53. 10.1016/j.pec.2012.09.005. 23068910 10.1016/j.pec.2012.09.005

[5] Deveugele M. Communication training: skills and beyond. Patient Educ Couns. 2015;98:1287–91. 10.1016/j.pec.2015.08.011. 26298220 10.1016/j.pec.2015.08.011

[6] Cushing AM. Learning patient centred communication: the journey and the territory. Patient Educ Couns. 2015;98:1236–42. 10.1016/j.pec.2015.07.024. 26297198 10.1016/j.pec.2015.07.024

[7] Makoul G. Essential elements of communication in medical encounters: the Kalamazoo consensus statement. Acad Med. 2001;76:390–3. 10.1097/00001888-200104000-00021. 11299158 10.1097/00001888-200104000-00021

[8] Makoul G, Noble L, Gulbrandsen P, van Dulmen S, for the Consensus Working Group. Reinforcing the humanity in healthcare: the Glasgow consensus statement on effective communication in clinical encounters. Patient Educ Couns. 2024;122:108158. 10.1016/j.pec.2024.108158. 38330705 10.1016/j.pec.2024.108158

[9] Silverman J, Kurtz S, Draper J. Skills for communicating with patients. 3rd ed. London: CRC; 2016. 10.1201/9781910227268.

[10] Rossi MG, Sarangi S. Communication skills, expertise and ethics in healthcare education and practice. Rivista Italiana Di Filosofia Del Linguaggio. 2021;15:106–22. 10.4396/2021060INT2. 10.1558/cam.24729 39916105

[11] Ury WA, Berkman CS, Weber CM, Pignotti MG, Leipzig RM. Assessing medical students’ training in end-of-life communication: a survey of interns at one urban teaching hospital. Acad Med. 2003;78(5):530–7. 10.1097/00001888-200305000-00019. 12742792 10.1097/00001888-200305000-00019

[12] Kurtz S, Draper J, Silverman J. Teaching and learning communication skills in medicine. 2nd ed. London: CRC; 2005. 10.1201/9781315378398.

[13] Baile WF, Buckman R, Lenzi R, Glober G, Beale EA, Kudelka AP. SPIKES—a six-step protocol for delivering bad news: application to the patient with cancer. Oncologist. 2000;5:302–11. 10.1634/theoncologist.5-4-302. 10964998 10.1634/theoncologist.5-4-302

[14] Ng SL, Kinsella EA, Friesen F, Hodges B. Reclaiming a theoretical orientation to reflection in medical education research: A critical narrative review. Med Educ. 2015;49:461–75. 10.1111/medu.12680. 25924122 10.1111/medu.12680

[15] Reeves S, Fletcher S, Barr H, Birch I, Boet S, Davies N, et al. A BEME systematic review of the effects of interprofessional education: BEME guide 39. Medi Teach. 2016;38:656–68. 10.3109/0142159X.2016.1173663. 10.3109/0142159X.2016.1173663 27146438

[16] Setyonugroho W, Kennedy KM, Kropmans TJB. Reliability and validity of OSCE checklists used to assess communication skills in health professionals: A systematic review. Patient Educ Couns. 2015;98:1482–91. 10.1016/j.pec.2015.06.004. 10.1016/j.pec.2015.06.004 26149966

[17] Newman LR, Lown BA, Jones RN, Johansson A, Schwartzstein RM, Feller-Kopman DJ. Developing a peer assessment of professionalism tool for students during clinical clerkship. Acad Med. 2009;84:574–81. 10.1097/ACM.0b013e31819fb3a7.19704189

[18] Bigi S. Argumentative discourse in clinical dialogues: an interdisciplinary perspective. Patient Educ Couns. 2025;133:108626. 10.1016/j.pec.2024.108626. 39740408 10.1016/j.pec.2024.108626

[19] von Fragstein M, Silverman J, Cushing A, Quilligan S, Salisbury H, Wiskin C, UK Council for Clinical Communication Skills Teaching in Undergraduate Medical Education. UK consensus statement on the content of communication curricula in undergraduate medical education. Med Educ. 2008;42:1100–7. 10.1111/j.1365-2923.2008.03137.x. 18761615 10.1111/j.1365-2923.2008.03137.x

[20] Rider EA, Keefer CH. Communication skills competencies: definitions and a teaching toolbox. Med Educ. 2006;40:624–9. 10.1111/j.1365-2929.2006.02500.x. 16836534 10.1111/j.1365-2929.2006.02500.x

[21] White SJ, Barello S, Cao di San Marco E, Colombo C, Eeckman E, Gilligan C, Graffigna G, Jirasevijinda T, Mosconi P, Mullan J, Rehman SU, Rubinelli S, Vegni E, Krystallidou D. Critical observations on and suggested ways forward for healthcare communication during COVID-19: pEACH position paper. Patient Educ Couns. 2021;104:217–22. 10.1016/j.pec.2020.12.025. 33419600 10.1016/j.pec.2020.12.025 PMC7833684

