# Supplementary material for: Mind the gap: a nationwide survey study of undergraduate medical communication curricula in Italy
Source: BMC Med Educ. 2026 Mar 9;26:619. doi: 10.1186/s12909-025-08522-8 (PMC13081256; doi:10.1186/s12909-025-08522-8)
Supplement: Supplementary file 1 — Supplementary Material 1. (Appendix A). [file 12909_2025_8522_MOESM1_ESM.docx]

**SUPPLEMENTARY MATERIALS – APPENDIX A**

**SURVEY**

**[For the original version in Italian, see below]**

**TITLE: “Survey on the state of communication teaching in medicine in Italy”**

**ONLINE SURVEY**(published on the EUSurvey platform)

**Information for Participants**

**Study Description**

This survey is conducted by researchers from the Interdepartmental Center for Ethics and Integrity in Research of the National Research Council (CNR), the Department of Linguistic Sciences and Foreign Literatures at Università Cattolica del Sacro Cuore in Milan, and the Department of Brain and Behavioral Sciences at the University of Pavia.

The aim of the survey is to map current academic practices of teaching communication in medicine and health professions in the Italian context. The questionnaire is intended for instructors who are currently responsible for a university course on this subject.

Completing the questionnaire takes approximately 15 minutes. If you teach more than one course, we kindly ask you to complete the questionnaire separately for each course.

### The Questionnaire

Your participation in the survey is entirely voluntary, and the questionnaire is anonymous.

If you decide to participate, you may still withdraw your consent at any time without providing any reason, simply by closing the browser window; no data will be recorded. However, once the form is fully completed and submitted, it will no longer be possible to delete your responses, as the questionnaire is anonymous and cannot be traced.

Consent does not involve the collection of personal data, as it is sufficient to click “YES” on the first screen to proceed with the questionnaire.

Given the expected sample size, there remains a residual risk of indirect identification of participants through secondary data analysis. Such analyses are beyond the scope of the survey, and no attempt will be made to identify participants, even indirectly. Moreover, if personal information of any kind is entered in open-text fields or comments, this information will not be retained to protect the anonymity of participants.

The information collected will be used solely for scientific purposes, such as statistical and qualitative analyses, presentations at conferences and seminars, and scientific publications.

By answering the questions and submitting your responses, you confirm that you have read and understood the information provided in this document and agree to participate in the study.

### The EUSurvey Platform

The “EUSurvey” platform, which hosts the questionnaire, complies with the provisions of the General Data Protection Regulation (GDPR).

The questionnaire is anonymous. During its completion, the only data processed are navigation-related data required to ensure the functionality and security of the platform and to prevent potential cyberattacks (technical and operational cookies). These navigation data are retained only for the time strictly necessary for these purposes.

The “anonymous survey mode” setting was selected during the creation of the questionnaire to ensure anonymous browsing.

For more information on data processing by the EUSurvey platform, you can consult the Privacy Policy (in Italian) at:

- <https://ec.europa.eu/eusurvey/home/privacystatement?language=it>;
- <https://ec.europa.eu/info/cookies_it>

### Contacts

This study was reviewed and approved by the Ethics and Integrity in Research Committee of the CNR.

For any information regarding this study, its results, or the measures taken to protect your anonymity, please contact Dr. Roberta Martina Zagarella at: roberta.zagarella@cnr.it

**Informed Consent**

By clicking “YES” you confirm that you have read and understood the information above and you give your consent to participate in the survey.

- Yes
- No

## **SECTION 1. INFORMATION ABOUT THE INSTRUCTOR AND THE COURSE**

**Are you currently in charge of an academic course in communication within the medical/healthcare field?**

- Yes
- No (if no, the survey ends here)

1. **Gender:**

- Female
- Male
- Prefer not to say

1. **Age:**

- < 30
- 31–40
- 41–50
- 51–66
- ≥ 67

1. **What is your current professional status?** *(Select only one option)*

- Full Professor
- Associate Professor
- Researcher
- Adjunct Professor
- Honorary/Emeritus Professor
- Other (please specify): ___________

1. **What is your academic discipline (GSD – Scientific Disciplinary Group)?**

**[A list of academic disciplinary groups (GSDs) is provided, along with the additional option “Not applicable”]**

1. **Which academic discipline is your medical communication course assigned to?**
   [Same **GSD** list applies, **with the additional option “I don't know"]**
2. **In which Region is the University offering your medical communication course located?**

- Abruzzo
- Basilicata
- Calabria
- Campania
- Emilia-Romagna
- Friuli-Venezia Giulia
- Lazio
- Liguria
- Lombardy
- Marche
- Molise
- Piedmont
- Apulia
- Sardinia
- Sicily
- Tuscany
- Trentino-Alto Adige
- Umbria
- Aosta Valley
- Veneto

1. **How many years have you been teaching communication in medicine?**

- Less than 1 year
- 1–5 years
- 6–10 years
- Over 10 years

1. **In which degree program do you teach medical communication?**

- Degree Program in Medicine
- Degree Program in Health Professions (please specify)
- Other (please specify)

1. **In which year of the program is your medical communication course scheduled?**

- First year
- Second year
- Third year
- Fourth year
- Fifth year
- Sixth year
- Other (please specify)

1. **Is your medical communication course:**

- Mandatory
- Optional

1. **Is your medical communication course:**

- A module within a broader course
- A stand-alone course

1. **In what language is your medical communication course taught?**

- Italian
- English
- Other (please specify)

1. **How many university credits (CFU) does your medical communication course carry?**

- 1
- 2
- 3
- Other (please specify)

1. **How long has your medical communication course been active?**

- 1–3 years
- 4–6 years
- 7–10 years
- More than 10 years
- I don’t know

1. **How did you acquire your specific skills in the field of medical communication?** *(Select all relevant options)*

- Formal academic training in medical communication (e.g., Master’s or Advanced Training Course)
- Non-academic certifications in medical communication
- Hands-on experience with patients
- Research experience in medical communication
- Other (please specify)

1. **Have you ever participated in training programs specifically for teaching communication in medicine?** (e.g., Train the Trainers programs)

- Yes
- No

1. **Are you or have you ever been a member of a scientific society operating in the field of medical communication?**

- Yes
- No

**17a**. **If yes, which one?**

- ______________________________

**SECTION 2: COURSE OBJECTIVES AND CONTENT**

**18. What are the primary objectives of your teaching on communication in medicine?** *(Select up to three relevant options)*

- To promote knowledge and understanding of core communication skills in medical practice (e.g., patient-centered communication)
- To encourage the development of attitudes that support good communication practices in medicine
- To highlight the ethical and deontological aspects of medical communication
- To enhance critical self-awareness and awareness of others in specific medical contexts
- To develop the ability to use appropriate communication skills in specific clinical settings (e.g., emergency care)
- To communicate with individuals from different sociocultural backgrounds
- To promote the conscious use of communication as a therapeutic act
- Other (please specify)

**19. What are the main topics covered in your course on communication in medicine?** *(Select the most relevant options, max 5)*

- Interprofessional communication among members of the healthcare team
- Communication with the patient during diagnosis and treatment
- Managing emotions during interactions with patients and caregivers
- Communication in emergency and crisis situations (e.g., emergency department)
- Communication in the management of chronic cases and long-term illnesses
- Communication with specific patient groups (e.g., patients with disabilities, psychiatric patients)
- Communication across different stages of the patient’s life (e.g., pediatrics, geriatrics)
- Communication in preventive medicine and health promotion
- Communication in delivering difficult news (e.g., severe diagnoses)
- Communication at the end of life
- Communication with couples in assisted reproduction
- Communication and genetic counseling
- Communication in culturally diverse contexts (cultural sensitivity)
- Communication in team-based learning environments
- Communication and decision-making in conflict situations
- Use of digital tools in medical communication (e.g., telemedicine)
- Communication in clinical research and scientific communication
- Ethics of communication and informed consent
- Legal aspects and privacy protection in medical communication
- Other (please specify)

**20.** **When thinking about communication, which of the following statements do you find most relevant to describe it?**

- - The transmission of information from a sender to a receiver
  - The production and exchange of meaning through languages
  - Everything that contributes to creating a relationship between two or more individuals
  - The ways in which one can influence the preferences or behaviors of an interlocutor
  - The realization of an empathetic relationship between two or more individuals
  - Other (please specify)

**21. Considering the various factors involved in communication with patients, which would be most useful to focus on in a teaching context?** *(max 3)*

- - The emotional dimension
  - The functions of gestures, posture, and eye contact
  - The pragmatic functions of linguistic structures
  - The dimension of implicit meaning
  - Cultural components
  - Medical language as a specialized language
  - Figurative uses of language
  - The functions of intonation
  - The time and setting of communication
  - Other (please specify)

**SECTION 3: TEACHING METHODS**

**22. Which teaching methods do you most frequently use in your instruction on communication in medicine?** *(Select up to 4 relevant options)*

- Lectures
- Group work
- Use of digital technologies (e.g., online platforms, apps)
- Discussion of clinical cases
- Analysis of videos of interactions between patients and healthcare professionals
- Guest lectures by external experts
- Other (please specify)

**23. Which methods do you most frequently use to train students in practical communication skills?** *(Select only relevant options, max 4)*

- - Classroom role-playing sessions
  - Discussion of real clinical cases
  - Collaboration with external professionals to provide hands-on experience in communication
  - Practical workshops on nonverbal communication
  - Use of narrative medicine techniques
  - Use of video-feedback to improve communication skills
  - Encouraging self-reflection on personal communication styles
  - Integrating mindfulness techniques to enhance communication awareness
  - Implementing mentoring sessions to guide students in developing communication skills
  - Providing detailed feedback on students’ communication performance
  - Simulating stressful communication scenarios
  - Use of individual or group coaching techniques
  - Other (please specify)

**24. How would you make the teaching of communication in medicine more consistent with other courses?** *(Select up to 3 priority options)*

- - Through practical simulations with professional actors
  - Using video recording tools to analyze and improve communication skills
  - Collaborating with medical professionals for hands-on experiences in hospitals or clinics
  - Incorporating real clinical cases into learning activities
  - Creating communication scenarios based on real medical situations
  - Involving “expert patients” in student training
  - Integrating medical communication with other medical disciplines (e.g., bioethics, psychology)
  - Using innovative technologies (e.g., virtual reality, AI, metaverse) for advanced simulations
  - Integrating problem-based learning (PBL) approaches
  - Other (please specify)

**SECTION 4: ASSESSMENT METHODS**

**25.** **Which methods do you use to assess the communication skills acquired by your students?** *(Select up to 3 relevant options)*

- - Written exams
  - Oral exams
  - Practical simulations (role-playing with simulated patients)
  - Peer-to-peer assessments
  - Group projects
  - Evaluations of real interactions with patients
  - Other (please specify)

**26. How do you assess the effectiveness of your course on communication in medicine?** *(max 3 options)*

- Through analysis of student evaluations of their learning experience
- By directly observing students’ communicative interactions during practical exercises
- Using specific tests or exams to assess communication skills acquired by students
- Conducting interviews or focus groups with students to gather detailed feedback on the course
- Other (please specify)

**SECTION 5: FUTURE DEVELOPMENTS**

**27.** **What improvements or developments would you like to implement in your teaching of communication in medicine?** *(Select up to 3 relevant options)*

- - Integration of new technologies
  - Greater involvement of external experts
  - Involvement of real patients
  - Continuous updating of course content
  - Expansion of practical training opportunities (e.g., internships)
  - Additional teaching resources (books, guides, etc.)
  - Availability of more credits/hours for the course
  - Relocating the course to a different year of the degree program
  - Other (please specify)

**28. Do you have any additional comments or suggestions you would like to share regarding the teaching of communication in medicine?**

- - Yes (please specify)
  - No

Thank you for your participation!

**TITOLO: “Indagine sullo stato dell’insegnamento della comunicazione in medicina in Italia”**

**SURVEY ONLINE**

(inserita sulla piattaforma EUSurvey)

**Informativa per i partecipanti**

**Descrizione dello studio**

Questa indagine è condotta da ricercatrici afferenti al Centro Interdipartimentale per l’Etica e l’Integrità nella Ricerca del CNR, al Dipartimento di Scienze Linguistiche e Letterature Straniere dell’Università Cattolica del Sacro Cuore di Milano e al Dipartimento di Scienze del Sistema Nervoso e del Comportamento dell’Università di Pavia.

L’obiettivo dell’indagine è effettuare una mappatura delle pratiche accademiche attuali di insegnamento di comunicazione in medicina e nelle professioni sanitarie nel contesto italiano. Il questionario è rivolto a docenti che attualmente sono incaricati di un corso universitario in questa materia.

Il questionario richiede circa 15 minuti per essere compilato. Qualora fosse titolare di più insegnamenti, Le chiediamo di compilare il questionario più volte (una volta per ciascun corso).

**Il questionario**

La Sua partecipazione all’indagine è interamente volontaria e il questionario è anonimo.

Se deciderà di partecipare, potrà comunque revocare il Suo consenso, senza fornire alcuna motivazione, semplicemente chiudendo la finestra di navigazione o il browser; nessun dato sarà registrato. Tuttavia, una volta compilata definitivamente e validata la scheda, non sarà più possibile cancellare le risposte che ha fornito, dal momento che il questionario è anonimo e quindi impossibile da individuare.

L’espressione del consenso non comporta una raccolta di dati personali poiché è sufficiente cliccare sull’opzione SI nella prima schermata per procedere con la compilazione del questionario.

In base alla numerosità attesa del campione, permane la possibilità residua di identificazione indiretta degli interessati a seguito di analisi secondarie sui dati rilevati. Tali analisi esulano dagli obiettivi dell’indagine e non verrà effettuato alcun tentativo di identificazione, anche indiretta, dei partecipanti. Inoltre, nel caso in cui, nelle caselle delle risposte aperte o dei commenti, venissero inserite informazioni personali di qualsiasi genere, tali informazioni non saranno trattenute, in modo da non mettere a rischio l’anonimato dei partecipanti.

Le informazioni raccolte saranno utilizzate unicamente per scopi scientifici, ad esempio per analisi statistiche, analisi qualitative, presentazioni a convegni e seminari, pubblicazioni scientifiche.

Rispondendo alle domande e inviando le Sue risposte, conferma di aver letto e compreso le informazioni fornite in questa informativa e accetta quindi di partecipare allo studio.

**La piattaforma EUSurvey**

La piattaforma “EUSurvey”, che ospita il questionario, è conforme alle disposizioni del “Regolamento generale sulla protezione dei dati” (il cosiddetto GDPR).

Il questionario è anonimo. Nel corso della compilazione del questionario, gli unici dati trattati sono quelli relativi alla navigazione, necessari a garantire la funzionalità e la sicurezza della piattaforma utilizzata e la prevenzione di possibili attacchi hacker (cookie tecnici ed operativi). Tali dati di navigazione saranno trattenuti solo per il tempo strettamente necessario a queste finalità.

L’impostazione “modalità indagine anonima” è stata da noi selezionata in fase di costruzione del questionario, in modo da garantire la navigazione in anonimato.

Per maggiori informazioni sul trattamento dei dati effettuato dalla piattaforma “EUSurvey” è possibile consultare la Privacy Policy in lingua italiana ai siti: <https://ec.europa.eu/eusurvey/home/privacystatement?language=it>; <https://ec.europa.eu/info/cookies_it>.

**Contatti**

L’indagine è stata revisionata e approvata dalla Commissione per l’Etica e l’Integrità nella Ricerca del CNR.

Per qualsiasi informazione su questo studio, sui risultati e sulle misure di tutela del Suo anonimato, può rivolgersi alla Dott.ssa Roberta Martina Zagarella, al seguente indirizzo email: [roberta.zagarella@cnr.it](mailto:roberta.zagarella@cnr.it).

**Consenso informato**

**Cliccando “SI” conferma di aver letto e compreso l’informativa fornita sopra e presta il Suo consenso a partecipare all’indagine.**

- Sì
- No

**SEZIONE 1. INFORMAZIONI SUL PROFILO DEL DOCENTE E SULL’INSEGNAMENTO**

**È attualmente titolare di un insegnamento accademico di comunicazione in ambito medico/sanitario?**

- - Sì
  - No (se no, la survey termina qui)

**1. Sesso:**

- - femmina
  - maschio
  - preferisco non rispondere

**2. Età:**

- - < 30
  - 31-40
  - 41-50
  - 51-66
  - Maggiore o uguale a 67

**3. Qual è il Suo attuale status professionale?** *(Seleziona una sola opzione)*

- - Professore Ordinario
  - Professore Associato
  - Ricercatore
  - Professore a Contratto
  - Professore Onorario/Emerito
  - Altro (specificare): ___________

**4. Quale è il Suo settore scientifico disciplinare (SSD)?**

[Menu a tendina con SSD riconosciuti al momento della somministrazione, più opzione “Non applicabile”]

**5. A quale settore scientifico disciplinare è attribuito il Suo insegnamento di comunicazione in medicina?**

[Menu a tendina con SSD riconosciuti al momento della somministrazione, più opzione “Non so”]

**6. In quale Regione si trova l’Ateneo dove eroga il corso di comunicazione in medicina?**

- - Abruzzo
  - Basilicata
  - Calabria
  - Campania
  - Emilia-Romagna
  - Friuli-Venezia Giulia
  - Lazio
  - Liguria
  - Lombardia
  - Marche
  - Molise
  - Piemonte
  - Puglia
  - Sardegna
  - Sicilia
  - Toscana
  - Trentino-Alto Adige
  - Umbria
  - Valle D’Aosta
  - Veneto

**7. Da quanti anni insegna comunicazione in medicina?**

- - Meno di 1 anno
  - 1-5 anni
  - 6-10 anni
  - Oltre 10 anni

**8. In quale corso di laurea eroga l’insegnamento di comunicazione in medicina?**

- - Corso di Laurea in Medicina
  - Corso di Laurea in Professioni Sanitarie (specificare quale): ___________
  - Altro (specificare): ___________

**9. A che anno del corso è collocato il Suo insegnamento di comunicazione in medicina?**

- - Primo anno
  - Secondo anno
  - Terzo anno
  - Quarto anno
  - Quinto anno
  - Sesto anno
  - Altro (specificare): ___________

**10. Il Suo insegnamento di comunicazione in medicina è:**

- - Obbligatorio
  - Opzionale

**11. Il Suo insegnamento di comunicazione in medicina è:**

- un modulo all’interno di un corso più ampio
- un corso a sé stante

**12. In che lingua è tenuto il Suo insegnamento di comunicazione in medicina?**

- - Italiano
  - Inglese
  - Altro (specificare): __________________

**13. A quanti CFU corrisponde il Suo insegnamento di comunicazione in medicina?**

- - 1
  - 2
  - 3
  - Altro (specificare): _________________

**14. Da quanti anni è attivo il Suo insegnamento di comunicazione in medicina?**

- 1-3
- 4-6
- 7-10
- Più di 10
- Non so

**15. Come ha acquisito le Sue competenze specifiche nel campo della comunicazione in medicina?** *(Selezionare tutte le opzioni pertinenti)*

- - Formazione accademica specifica in comunicazione medica (ad es. Master o Corso di Perfezionamento)
  - Certificazioni non accademiche in comunicazione medica
  - Esperienza pratica sul campo con i pazienti
  - Esperienza nella ricerca sulla comunicazione medica
  - Altro (specificare): ______________

**16. Ha mai partecipato a programmi di formazione specifici per l’insegnamento della comunicazione in medicina? (Ad es. programmi di *Train the trainers*)**

- - Sì
  - No

**17. È stato o è attualmente iscritto ad una società scientifica operante nell’ambito della comunicazione medica?**

- - Sì
  - No

**17a. Se sì, quale?**

- ______________________________

**SEZIONE 2: GLI OBIETTIVI E I CONTENUTI DEL CORSO**

**18. Quali sono gli obiettivi prioritari del Suo insegnamento di comunicazione in medicina?** *(Selezionare al massimo tre opzioni pertinenti)*

- - Promuovere la conoscenza e la comprensione delle abilità comunicative centrali nella pratica medica (es. patient-centred communication)
  - Stimolare lo sviluppo di atteggiamenti favorevoli a buone pratiche di comunicazione in medicina
  - Mettere in luce gli aspetti etico-deontologici della comunicazione medica
  - Incrementare la consapevolezza critica di sé e degli altri in specifici contesti medici
  - Sviluppare la capacità di utilizzare competenze comunicative appropriate in specifici setting clinici (es. emergenza-urgenza)
  - Comunicare con interlocutori appartenenti a diversi contesti socioculturali
  - Promuovere l’utilizzo consapevole della comunicazione come atto terapeutico
  - Altro (specificare): ________________

**19. Quali sono i principali argomenti trattati nel Suo corso di comunicazione in medicina?** *(Selezionare le opzioni più pertinenti, max 5)*

- - Comunicazione interprofessionale tra membri del team medico
  - Comunicazione con il paziente durante la diagnosi e il trattamento
  - Gestione delle emozioni durante le interazioni con i pazienti e i caregivers
  - Comunicazione in situazioni di emergenza e crisi (es. pronto soccorso)
  - Comunicazione nella gestione dei casi cronici e delle malattie a lungo termine
  - Comunicazione con gruppi di pazienti specifici (es. pazienti con disabilità, psichiatrici)
  - Comunicazione attraverso diverse fasi della vita del paziente (ad es. pediatria, geriatria)
  - Comunicazione nell’ambito della medicina preventiva e promozione della salute
  - Comunicazione nella divulgazione di notizie difficili (es. diagnosi gravi)
  - Comunicazione nel fine vita
  - Comunicazione con la coppia nella procreazione assistita
  - Comunicazione e counseling genetico
  - Comunicazione in contesti culturalmente diversificati (*cultural sensitivity*)
  - Comunicazione in contesti di team-based learning
  - Comunicazione e decisione in situazioni di conflitto
  - Utilizzo di strumenti digitali nella comunicazione medica (es. telemedicina)
  - Comunicazione nell’ambito della ricerca clinica e comunicazione scientifica
  - Etica della comunicazione e consenso informato
  - Aspetti legali e rispetto della privacy nella comunicazione medica
  - Altro (specificare): _________________

**20. Pensando alla comunicazione, quale delle seguenti affermazioni Le sembra più pertinente per descriverla?**

- - La trasmissione di informazioni da un mittente a un destinatario
  - La produzione e lo scambio di significati attraverso le lingue
  - Tutto ciò che concorre a creare una relazione tra due o più individui
  - I modi in cui è possibile influire sulle preferenze o i comportamenti di un interlocutore
  - Il realizzarsi di una relazione empatica tra due o più individui
  - Altro (specificare): ____________________

**21. Pensando ai vari fattori che entrano in gioco nella comunicazione con il paziente, su quali sarebbe più utile soffermarsi in un insegnamento?** *(max 3)*

- - La dimensione emotiva
  - Le funzioni dei gesti, della postura e dello sguardo
  - Le funzioni pragmatiche delle strutture linguistiche
  - La dimensione dell’implicito
  - Le componenti culturali
  - La lingua della medicina come lingua speciale
  - Gli usi figurati del linguaggio
  - Le funzioni dell’intonazione
  - Il tempo e il setting della comunicazione
  - Altro (specificare): ______________________

**SEZIONE 3: METODOLOGIE DIDATTICHE**

**22. Quali metodologie didattiche adotta con maggiore frequenza nel Suo insegnamento della comunicazione in medicina?** *(Indicare max 4 opzioni pertinenti)*

- - Lezioni frontali
  - Lavori di gruppo
  - Utilizzo di tecnologie digitali (es. piattaforme online, app)
  - Discussione di casi clinici
  - Analisi di video di interazioni tra pazienti e operatori sanitari
  - Testimonianze di esperti esterni
  - Altro (specificare): ___________

**23. Quali metodologie adotta più frequentemente per esercitare gli studenti nelle competenze comunicative pratiche?** *(Selezionare solo le opzioni pertinenti, max 4)*

- - Organizzazione di sessioni di role-playing in aula
  - Discussione di casi clinici reali
  - Collaborazione con professionisti esterni per fornire esperienze pratiche sul tema della comunicazione
  - Organizzazione di laboratori pratici dedicati alla comunicazione non verbale
  - Utilizzo di tecniche di medicina narrativa
  - Utilizzo di video-feedback per migliorare le abilità comunicative
  - Promozione dell’auto-riflessione sugli stili comunicativi personali
  - Integrazione di tecniche di mindfulness per favorire la consapevolezza comunicativa
  - Implementazione di sessioni di mentoring per guidare gli studenti nello sviluppo delle competenze comunicative
  - Feedback dettagliato sulle performance degli studenti in ambito comunicativo
  - Simulazione di situazioni di stress in ambito comunicativo
  - Utilizzo di tecniche di coaching individuali o di gruppo
  - Altro (specificare): ___________

**24. Come renderebbe l’insegnamento della comunicazione in medicina più coerente con altri corsi?** *(Selezioni fino a 3 opzioni per Lei prioritarie)*

- - Attraverso simulazioni pratiche con attori professionisti
  - Utilizzando strumenti di registrazione video per analizzare e migliorare le abilità comunicative
  - Collaborando con professionisti del settore medico per esperienze pratiche in ospedali o cliniche
  - Incorporando casi clinici reali nelle attività di apprendimento
  - Creando scenari di comunicazione basati su situazioni mediche reali
  - Coinvolgendo nella formazione degli studenti “pazienti esperti”
  - Integrando la comunicazione in medicina con altre discipline mediche (es. bioetica, psicologia)
  - Utilizzando tecnologie innovative (es. realtà virtuale, AI, metaverso) per simulazioni avanzate
  - Integrando approcci di apprendimento basati su problemi (PBL)
  - Altro (specificare): ___________

**SEZIONE 4: METODI DI VALUTAZIONE**

**25. Quali metodi utilizza per valutare le competenze comunicative apprese dai Suoi studenti?** *(Selezioni max 3 opzioni pertinenti)*

- - Esami scritti
  - Esami orali
  - Simulazioni pratiche (role-playing con pazienti simulati)
  - Valutazioni peer-to-peer
  - Progetti di gruppo
  - Valutazioni di interazioni reali con i pazienti
  - Altro (specificare): ___________

**26. Come valuta l’efficacia del Suo corso di comunicazione in medicina?** *(max 3 opzioni)*

- - Attraverso l’analisi delle valutazioni degli studenti sulla loro esperienza di apprendimento
  - Mediante l’osservazione diretta delle interazioni comunicative degli studenti durante le esercitazioni pratiche
  - Utilizzando test o esami specifici per valutare le competenze comunicative acquisite dagli studenti
  - Conducendo interviste o focus group con gli studenti per raccogliere feedback dettagliati sul corso
  - Altro (specificare): ____________

**SEZIONE 5: SVILUPPI FUTURI**

**27. Quali miglioramenti o sviluppi vorrebbe implementare nel Suo insegnamento di comunicazione in medicina?** *(Selezioni max 3 opzioni pertinenti)*

- - Integrazione di nuove tecnologie
  - Maggior coinvolgimento di esperti esterni
  - Coinvolgimento di pazienti reali
  - Aggiornamento continuo dei contenuti
  - Espansione delle opportunità di formazione pratica (es. tirocinio)
  - Ulteriori risorse didattiche (libri, guide, ecc.)
  - Disponibilità di un numero maggiore di crediti/ore per il corso
  - Collocazione del corso in un anno del corso di studi diverso da quello attuale
  - Altro (specificare): ___________

**28. Ha ulteriori osservazioni o suggerimenti che desidera condividere in merito all’insegnamento della comunicazione in medicina?**

- - Sì (specificare): ___________
  - No

Grazie per la Sua partecipazione!
